# Hydrogels for Cardiac Tissue Regeneration: Current and Future Developments

**DOI:** 10.3390/ijms26052309

**Published:** 2025-03-05

**Authors:** Sonja Holme, Stephen M. Richardson, Jordi Bella, Christian Pinali

**Affiliations:** 1 Division of Cell Matrix Biology & Regenerative Medicine, School of Biological Sciences, Faculty of Biology, Medicine and Health, University of Manchester, Manchester M13 9PT, UK; sonja.holme@manchester.ac.uk (S.H.); s.richardson@manchester.ac.uk (S.M.R.); 2 Division of Cardiovascular Sciences, School of Medical Sciences, Faculty of Biology, Medicine and Health, University of Manchester, Manchester M13 9NT, UK

**Keywords:** myocardial infarction, hydrogel, extracellular matrix, myocardial tissue engineering

## Abstract

Myocardial infarction remains a leading cause of death worldwide due to the heart’s limited regenerative capability and the current lack of viable therapeutic solutions. Therefore, there is an urgent need to develop effective treatment options to restore cardiac function after a heart attack. Stem cell-derived cardiac cells have been extensively utilised in cardiac tissue regeneration studies. However, the use of Matrigel as a substrate for the culture and maturation of these cells has been a major limitation for the translation of this research into clinical application. Hydrogels are emerging as a promising system to overcome this problem. They are biocompatible and can provide stem cells with a supportive scaffold that mimics the extracellular matrix, which is essential for repairing damaged tissue in the myocardium after an infarction. Thus, hydrogels provide an alternative and reproducible option in addressing myocardial infarction due to their unique potential therapeutic benefits. This review explores the different types of natural and synthetic polymers used to create hydrogels and their various delivery methods, the most common being via injection and cardiac patches and other applications such as bioprinting. Many challenges remain before hydrogels can be used in a clinical setting, but they hold great promise for the future of cardiac tissue regeneration.

## 1. Introduction

**Figure 1:**
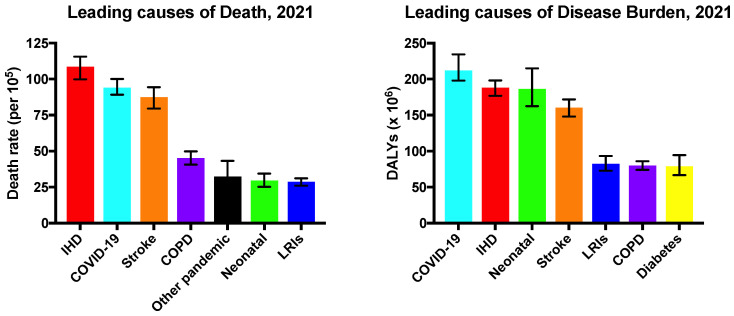
Leading causes of death and disease burden worldwide in 2021. Mortality is shown as the age-standardised death rate per 100,000. Disease burden is measured in the number of disability-adjusted life years (DALYs) in millions. IHD, ischemic heart disease; COPD, chronic obstructive pulmonary disease; LRIs, lower respiratory infections. Other pandemic-related mortality includes excess deaths associated with the COVID-19 pandemic. Data from the Global Burden of Disease 2021 study [3].

Cardiovascular disease (CVD) affects the heart and blood vessels [1]. CVD is the leading cause of death worldwide and contributes to an overall decline in the health of the population. It is estimated that, in 2022, 19.8 million deaths were linked to CVD [2]. Comprehensive epidemiological data from the Global Burden of Disease 2021 study [3] indicate that ischemic heart disease (IHD), which falls within the spectrum of CVD, surpassed the COVID-19 pandemic as the direct leading cause of death around the world, and was the second leading cause of disease burden (Figure 1). It is estimated that approximately 250 million people worldwide are living with IHD and that about 9 million deaths are caused each year by this disease, 13% of the global annual mortality [3]. The prevalence of IHD globally is on an upward trend and is forecasted to continue to increase [1,3].

A major cause of the overall increase in CVD is the ageing and growth of the population worldwide [4]. This issue warrants significant attention, as projections by the World Health Organisation indicate that the population over 60 will rise from 1 billion in 2019 to 1.4 billion by 2030, an increase of 40%. This upward trajectory is expected to continue, reaching 2.1 billion by 2050, marking a 50% increase [3,5,6]. As the population ages the prevalence and death toll associated with IHD and CVD are projected to increase. Therefore, it is becoming urgent to develop new therapeutic methods for cardiac tissue regeneration. In this review we discuss the use of hydrogels as substrates for the culture of stem cells and their differentiation into cardiac cells. We summarise the different types of natural and synthetic polymers used to create hydrogels, and discuss the most commonly used applications, injectable hydrogels, and hydrogel-based cardiac patches. Although many challenges still remain before the widespread clinical application of hydrogels, we can conclude that these materials hold great promise for the future of cardiac tissue regeneration.

## 2. Myocardial Infarction

Myocardial infarction (MI), commonly known as a heart attack, occurs predominantly due to a lack of blood flow to the heart. This typically arises from a blockage in the coronary arteries, which are responsible for supplying blood to the heart’s muscular tissue [7]. The obstruction may stem from a blood clot or the accumulation of fatty deposits known as plaque, leading to a condition referred to as atherosclerosis. When blood circulation to the heart’s muscle is obstructed, it results in the death of cardiac muscle cells [8]. MI can be fatal or lead to lifelong health complications. The heart has limited regenerative capabilities, so when the cardiac muscle cells die, the heart compensates by activating fibroblasts, this leads to fibrosis and the formation of scar tissue and over time weakens the heart’s ability to pump blood efficiently, eventually leading to heart failure [9]. Other MI complications involve arrhythmia, cardiogenic shock, stroke, heart valve problems, and the increased risk of another MI occurring [9,10]. An analysis of Swedish national registries examining 97,254 patients who survived an MI one week after discharge showed that within 365 days, 18.3% of the patients suffered a further cardiovascular event: a stroke, reoccurring MI, or cardiovascular death. Furthermore, of the patients who did not experience a cardiovascular event in this period, 20% would experience one or more within the following 3 years [11], highlighting the significant long-term risk to patients post MI.

### Current Treatments for MI

The current treatment options available for MI are devised to restore blood flow to the heart as fast as possible to minimise tissue damage and prevent any future complications. These approaches range from pharmacological options such as tissue plasminogen activator (tPA), which dissolves clots [12], to other medications, such as ACE inhibitors, calcium channel blockers, and beta-blockers, which help manage hypertension and decrease the heart’s need for oxygen. Non-invasive surgical options include percutaneous coronary intervention (PCI), which reduces the narrowing or blockage of the coronary artery [13]. However, in serious cases, a pacemaker or even a heart transplant may be required [12]. Despite advancements in healthcare, MI remains the leading cause of death globally [2]. Regenerative medicine holds a promising approach for the treatment and management of the complications caused by MI. Research efforts have focussed on therapeutic interventions such as biocompatible hydrogels that provide a supportive scaffold to the damaged area and promote tissue repair. Alongside this, stem cells can replace damaged cardiomyocytes with healthy ones [14]. The goal of these approaches is to promote cardiac tissue repair, regeneration, and the restoration of cardiac function following MI.

## 3. Development of Novel Therapies for Cardiac Regeneration

The heart has very limited regenerative capabilities. Cardiac myocytes (CM) proliferation does occur in the adult mammalian heart but the rate of replacement with new cells is less than 1% annually [15,16], which is inadequate to replace the millions of CMs lost in infarcted heart tissue. Research is striving to find a way to stimulate heart regeneration, to treat the symptoms caused by heart disease and MI, or to prevent them from even occurring [17]. Cell culture is a key approach for researching the biological mechanisms of cells, tissues, and organs [18]. The inner physiological mechanisms of organs are very complex, making it difficult for two-dimensional (2D) cell cultures to accurately represent their properties and biomechanics in vivo [19]. Thus, research has been directed towards developing three-dimensional (3D) cell culture systems, with the goal of replicating the organ’s internal processes, including the complex interactions between the cells and their extracellular environment [20,21]. Specifically, cardiac tissue contains cells with the ability to contract such as CMs and smooth muscle cells, alongside non-contractile cells like fibroblasts, endothelial cells, and leukocytes. These cells interact with each other in a specific manner that is organised to form an intricate 3D structure, crucial for the heart to function [22]. While 2D cultures are still extensively used in the cardiovascular field and possess many benefits for in vitro research, 3D cell culture systems offer a more realistic environment that can produce cardiac-like structures, which better represent the complex cell interactions as seen in vivo [20].

### 3.1. Cell-Based Therapies

Cell transplantation is one of the most promising fields currently being studied in cardiac tissue regeneration. Stem cell therapy involves generating new CMs following an MI to replenish the lost tissue in the heart [17]. Stem cells are defined as undifferentiated cells with the capacity to endlessly self-renew and can be programmed to differentiate into a variety of different cell types [21] (Table 1). They can be derived from multiple sources such as the placenta, umbilical cord, bone marrow, adipose tissue, and dental pulp [22,23]. By using specific growth factors, these stem cells can be differentiated into different types of cells such as smooth muscle cells, fibroblasts, endothelial cells, or CMs [22]. Adult stem cells, sometimes referred to as somatic stem cells, can be classified via the tissue they came from such as skeletal myoblasts and mesenchymal stem cells [24] (Table 1).

#### 3.1.1. Skeletal Myoblasts

Skeletal myoblasts are progenitor cells that originate in skeletal muscle. They were the first type of cell to be studied for cardiac tissue regeneration because of their ability to regenerate muscle tissue following an injury [25]. These cells have many attractive properties, including easy isolation, quick in vitro expansion, and the capacity to resist ischemia [26]. Given all this, their potential was considered for studying the remuscularization of damaged heart tissue [34] and has been thoroughly researched in vivo [26]. In heart damage because of an MI, the use of skeletal myoblasts successfully prevented remodelling in the left ventricle (LV) while preserving its pressure and ejection fraction [26,34]. Additionally, the contractility of the myocardium was maintained, and the damaged heart saw an improvement in compliance [35]. Furthermore, skeletal myoblasts were the first to be used in a clinical setting. In 2000, a 72-year-old male was the first patient who was given autologous skeletal myoblasts alongside a coronary artery bypass. The patient had been diagnosed with heart failure as a result of a severe MI. A 5-month follow-up showed an increase in left ventricle ejection fraction (LVEF) and an overall improvement in his symptoms of heart failure [36,37]. This study opened the way for multiple isolated cases and further clinical trials to be pursued [35]. While these studies show the promise of using skeletal myocytes as a stem cell therapy, the majority of the clinical trials were performed alongside a coronary artery bypass or a left ventricular assist device which could have skewed the results [35,37]. As such, it is still unclear how effective the use of skeletal myocytes is in cardiac regeneration and further investigation is needed to fully determine the effectiveness of using skeletal myoblasts as a stem cell treatment for cardiac repair.

#### 3.1.2. Mesenchymal Stem Cells

Mesenchymal stem cells (MSCs) are adult stem cells that have gained substantial interest in the field of regenerative medicine [26]. These cells are multipotent and can be derived from a range of tissues, most commonly bone marrow and adipose tissue, making them readily available [27]. MSCs can be differentiated into a variety of different cell types, including cardiomyocytes [26]. However, the results of using MSCs for cardiac regeneration have been mixed. Preclinical studies showed that transplanting MSCs into the heart after damage from an MI resulted in a marked improvement in cardiac function. On the other hand, the findings in multiple clinical trials showed that transplanting MSCs resulted in very limited help for patients with heart injuries [38,39,40]. There is still interest in investigating the potential of adult stem cells as a cell-based therapy. However, this potential has been obstructed by multiple limitations such as low cell retention, poor cell survival, and engraftment [27].

#### 3.1.3. Embryonic Stem Cells

Embryonic stem cells (ESCs) were first discovered in mouse embryonic cells obtained from the inner mass of blastocytes [28]. Subsequently, scientists identified human embryonic stem cells (hESCs) that can differentiate into all cell types and have the capacity for unlimited self-renewal. This discovery sparked great interest in the use of hESCs in tissue repair and organ regeneration [29]. However, their capacity to divide into any cell type is also a limitation as it can lead to the formation of cancerous tumours, making hESCs inappropriate for clinical use [30]. ESCs are also subject to ethical and political controversy, as the method of isolating these cells requires human embryos to be destroyed. People’s moral or religious beliefs question whether it is ethical to use human embryos, as they believe that an embryo represents a fully developed human [37]. However, this point of view is not shared by the entire population, which opens up debates and criticisms.

Focusing on research in cardiac repair, multiple studies have shown that CMs derived from hESCs can provoke arrhythmia after transplantation [41,42]. Nevertheless, there is contrasting evidence from Liu et al., who demonstrated remuscularization in the damaged heart tissue of macaque monkeys with hESC-CM grafts in a 2018 study [43]. These grafts significantly improved cardiac function in an MI model; no teratomas were detected, and only one monkey suffered from minor arrhythmias. This study used a higher than standard dose of embryonic stem cells that fully differentiated into cardiomyocytes, replacing progenitor cells, which may have contributed to the difference in results when compared to the previous studies mentioned. The authors did acknowledge that the arrhythmias observed could be linked to the hESC-CM grafts [43]. Although there was a significant decrease in the number of arrhythmias in this study, it still calls into question the possibility of them arising in human trials using this protocol. Due to the safety and ethical concerns of ESCs, further research is still required to determine their potential in cell-based therapies for treating MI.

#### 3.1.4. Induced Pluripotent Stem Cells

In 2006, induced pluripotent stem cells (iPSCs) were pioneered by Yamanaka’s group through the discovery of four transcription factors: SRY-box 2 (SOX2), Krüppel-like factor (Klf4), V-myc myelocytomatosis viral oncogene homolog (c-Myc), and octamer binding transcription factor 4 (Oct-4) [31]. They demonstrated that the forced expression of these transcription factors in mouse embryonic fibroblasts or adult mouse fibroblasts could revert the cells into a pluripotent state, enabling them to differentiate into all three germ layers: ectoderm, endoderm, and mesoderm [14]. Subsequently, they showed that this method could also be used to generate human induced pluripotent stem cells (hiPSCs) [32]. This discovery led to an alternative source of human stem cells that does not present the ethical complications of ESCs [33]. Additionally, hiPSCs have an even lower risk of immune rejection than other stem cells as they can be directly procured from each patient for individualised therapy [33,44]. One significant drawback of hiPSCs is their lack of maturity, which can cause issues after transplantation. The incorporation of the immature CMs derived from hiPSCs with the native adult CMs can disrupt electrical signalling, leading to an increased risk of arrhythmias [33,45]. In a 2016 study, cynomolgus monkeys were used to investigate the effects of injecting iPSC-CMs into the heart 14 days post MI [46]. The results revealed that the injected iPSC-CMs exhibited a survival period of 12 or more weeks and established electrical connections with the myocardium. Moreover, cardiac function and contractility showed improvement, alongside some remuscularisation of the damaged heart. Non-fatal arrhythmias were initially observed, possibly caused by the immaturity of the iPSC-CMs and their incomplete integration into the heart. However, by day 14, the frequency of these arrhythmias had started to decline, which may be attributed to the iPSC-CMs progressively maturing, improving their electrical integration into the heart [46]. Further research is being directed at developing strategies to facilitate the maturation of iPSC-CMs, among them incorporating biomaterials or fatty acids, using electrical stimulation, and increasing the culture time of the iPSCs [45,47]. Other studies have demonstrated that embedding hiPSC-CMs into an epicardial sheet and then transplanting the sheet onto the heart of an MI Porcine model showed improved left ventricular ejection fraction, contractility, and cardiac function [48,49]. While cardiac differentiation protocols are highly effective in generating hiPSC-CMs, the rate of differentiation may not always be 100% and any remaining undifferentiated iPSCs cells could cause tumour formation [50,51]. This highlights the potential iPSCs have in cardiac tissue regeneration. Nevertheless, there are still many challenges that need to be resolved before they can be effectively used for clinical application, especially those related to the delivery medium.

## 4. Application of Biomaterials to Enhance Cell-Based Regenerative Therapies

Matrigel is a basement-membrane matrix that contains many proteins found in the extracellular matrix (ECM). The ECM itself is a highly complex structure composed of various proteins, glycoproteins, proteoglycans, and glycosaminoglycans. These components are crucial for maintaining the plasticity and strength of the ECM [52]. The cardiac ECM regulates cellular growth and provides a supporting environment for cardiac cells such as CMs, vascular cells, fibroblasts, and inflammatory cells [53,54]. Replicating the ECM in cell culture is essential to creating a supportive environment to facilitate the differentiation and maturation of cardiac cells [54]. In this regard, Matrigel is widely considered the gold standard for cell culture as it mimics the roles of ECM in promoting cell growth and differentiation [55]. Collagen type IV, perlecan, laminin, and entactin are found in Matrigel alongside other basement membrane constituents, including proteases and growth factors [56], which play a crucial role in its function. However, there are major drawbacks of Matrigel, such as its undefined composition and batch-to-batch variation. Over 14,000 unique peptides and around 2000 proteins have been detected in Matrigel, and proteomic analyses show significant variability between batches [55]. In addition, as it is derived from mice, it can contain xenogenic contamination, further limiting its therapeutic potential [57]. It is also important to consider the ethical implications of its source, as Matrigel is produced from the Engelbreth–Holm–Swarm mouse tumour, an aggressive form of cancer that is induced in mice. This process causes significant suffering and distress to the animals as they endure the growth of the tumour, highlighting the importance of an alternative animal-free cell culture substrate [56]. All these limitations make Matrigel limited for clinical applications in cardiac tissue regenerative medicine. Thus, there is a need for the development of natural and synthetic biomaterials, such as hydrogels, to be used as alternative substrates for cell culture, growth, and differentiation.

### 4.1. Hydrogels

Hydrogels are three-dimensional networks generated via the crosslinking of hydrophilic polymers, which have the capability of absorbing and retaining large amounts of water causing the hydrogel to swell [58,59,60]. Hydrogels are used as scaffolds to provide support for cell differentiation and maturation by partially mimicking the properties of the ECM [61,62]. There are two broad types of hydrogels: natural and synthetic. Natural hydrogels are derived from naturally occurring polymers such as collagen, fibrin, and gelatin. Synthetic hydrogels are produced from synthetic polymers like polyvinyl alcohol and polyethylene glycol [60].

### 4.2. Hydrogel Crosslinking

Hydrogel formation may involve different crosslinking mechanisms, developing physical or chemical bonds between polymer chains that create a three-dimensional network [63]. Crosslinking plays a crucial role in the generation of hydrogels as it influences their biological, mechanical and chemical properties [64,65]. The crosslinks, whether physical or chemical, affect the elasticity, degradability, porosity and strength of the hydrogel [66]. The distinction between chemical and physical crosslinking lies in the nature of the bonds that hold the hydrogel together [66,67]. Physical crosslinking involves interactions such as van der Waals forces, hydrogen bonding, or ionic interactions and crystallite formation [68], whereas chemical crosslinking involves covalent bonds formed between the polymer chains, resulting in a stronger, stable and permanent network.

#### 4.2.1. Physical Crosslinking

Physical crosslinking can be reversible, meaning that the hydrogels can respond to environmental changes such as pH levels, temperature, or ionic strength [69]. Physical crosslinking is also biocompatible and less toxic, making it desirable in biomedical applications. Self-assembling hydrogels form spontaneously noncovalent bonds like van der Waals forces, hydrophobic interactions, and hydrogen bonds [70]. Individual hydrogen bonds are relatively weak interactions but when multiple hydrogen bonding occurs it can form the intricate networks characteristic of hydrogels. Natural hydrogel-forming polymers such as collagen, starch, agar, and gelatin can build extensive hydrogen bonding networks [60,69,71]. However, hydrogels that are crosslinked via hydrogen bonding alone have disadvantages such as weak mechanical strength. As such, hydrogen bonding can be combined with other methods of crosslinking to generate mechanically stronger hydrogels [71]. Hydrophobic interactions lead to significant water absorption and swelling, essential for the formation of hydrogel structures. Hydrogels made from chitosan, for example, are typically prepared using this method of hydrophobic interaction [60,72]. Hydrogels can also be formed from ionic and electrostatic interactions, which occur between oppositely charged groups by electrostatic force [71]. Alginate, a polysaccharide containing mannuronic and glucuronic acid, forms crosslinks with divalent cations such as calcium (Ca^2+^), magnesium (Mg^2+^), and barium (Ba^2+^). These cations bind to alginate’s glucuronate blocks, creating gel structures that can be used in biomedical applications such as drug delivery, wound healing, and tissue engineering [73]. While ionic–electrostatic interactions enable the hydrogels to self-repair, allowing them to reform after high stress, this crosslinking method limits the mechanical strength of the hydrogels [65]. A physical crosslinking technique widely used for both natural and synthetic polymers is the freeze–thawing method [74]. As seen in the production of poly vinyl alcohol (PVA) this process leads to the formation of crystallites acting as crosslinking sites in the polymer matrix eventually forming hydrogel structures [75].

#### 4.2.2. Chemical Crosslinking

Chemical crosslinking in hydrogels involves the formation of covalent bonds between polymer chains [76]. These bonds can be generated by incorporating functional monomers into the polymer matrix, or by joining together the carboxyl (-COOH), hydroxyl (-OH), or amino (-NH2) groups of polymer chains via crosslinking agents such as aldehydes [77]. These reactions form stable covalent bonds that define the structure and properties of the hydrogel network. Nevertheless, aldehydes are toxic, affecting the hydrogel’s biocompatibility [76]. Enzymatic crosslinking is an alternative approach in which enzymes such as tyrosinase, peroxidase or lysyl oxidase catalyse the crosslinking within polymer chains [77]. This is more advantageous as it is highly selective and only requires mild catalytic reactions without the need for toxic crosslinking agents [78]. Enzymatic crosslinking has facilitated the production of hydrogels sourced from various proteins, primarily collagen or gelatin. Additionally, it has become increasingly popular for injectable hydrogels due to their biocompatibility and ability to function under physiological conditions [79]. Enzymes typically function under conditions found in living organisms and, unlike many chemicals, are generally considered to be biologically compatible.

### 4.3. Hydrogels: Natural vs. Synthetic Polymers

There are two broad categories of hydrogels: natural and synthetic (Table 2 and Table 3). Natural hydrogels are derived from naturally occurring polymers such as collagen, fibrin, and gelatin and are highly biocompatible. Synthetic hydrogels are produced from synthetic polymers like polyvinyl alcohol and polyethylene glycol, possessing controllable and strong mechanical properties. These traits make both natural and synthetic hydrogels very attractive for cardiac tissue engineering [80].

### 4.4. Hybrid Hydrogels

Natural and synthetic polymers both have their own unique advantages. Natural biomaterials are highly biocompatible and have a greater affinity for cells. However, they tend to have a weaker mechanical strength and degrade at a faster rate than synthetic polymers. On the other hand, synthetic polymers offer stronger mechanical properties than natural polymers but may not be as biocompatible [97]. To combine the strengths of both, hybrid hydrogels have been developed [97,100]. Gelatin methacryloyl (GelMA) is a hybrid hydrogel developed by combining methacrylic anhydride with gelatin [101]. It has gained popularity in cardiac tissue engineering due to its biocompatibility, high cell adhesion, and the ability to regulate its biodegradability [102]. In addition, the stiffness and strength of the hydrogel can be increased by adjusting the amount of methacrylic anhydride [103]. Recent and ongoing research into hybrid hydrogels has shifted its focus towards the incorporation of conductive nanomaterials, such as gold nanomaterials, graphene oxide (GO), or carbon nanotubes (CNTs). As the heart relies on electrical signals to function and coordinate heartbeats, the intrinsic electrically conductive properties of these materials can greatly enhance hydrogels for cardiac tissue regeneration [104,105]. For example, Li et al. incorporated polydopamine-reduced graphene oxide (PDA/rGO) into GelMA hydrogels. CMs were then seeded into this modified hydrogel, which showed an increase in cell survival rate and faster maturation compared to GelMA hydrogel alone. Moreover, CMs in the PDA/rGO hydrogel group exhibited an accelerated beating rate, attributed to the increased conductivity resulting from the addition of PDA/rGO [106].

### 4.5. Self-Assembling Peptide Hydrogels

While hybrid hydrogels are a promising avenue for cardiac tissue regeneration, synthetic peptides have gained significant attention as they show properties of both natural macromolecules and synthetic polymers. To further advance tissue engineering, self-assembling peptide hydrogels (SAPHs) have been developed [107]. SAPHs represent a category of soft materials made from peptides and amino acids that function as fundamental building blocks. These hydrogels can entrap water or fluids within their molecular framework, transforming them into nanoscale hydrogel structures [94]. Utilising specific methods, peptides can be programmed to spontaneously self-assemble in aqueous conditions such as temperature, ionic strength, or pH [107]. SAPHs use various highly specific intermolecular interactions, such as hydrogen bonding, π-π stacking, electrostatic, ionic, hydrophobic, and van der Waals interactions, leading to the formation of different secondary structures such as α-helices and β-sheets [107,108]. These hydrogels are very tuneable and can be easily modified; they also provide ECM-like microenvironments, biocompatibility, and biodegradability, making them ideal for cardiac tissue engineering applications [109]. In a previous study, a SAPH modified with folic acid was created to differentiate iPSCs into cardiomyocytes in the hearts of mice that had suffered from an MI. The results showed that this modified SAPH enhanced the differentiation and survival of the iPSCs, leading to a reduction in fibrosis and increased cardiac function [110]. Another study by Burgess et al. investigated the cell adhesion functionalisation of the self-assembling peptides FEFEFKFK (F-phenylalanine; E: glutamic acid; K: lysine). This peptide can self-assemble into transparent and stable hydrogels [111,112,113]. To enhance its cell adhesion properties, they added the motif RGD (R: arginine; G: glycine; D: aspartic acid). Using the peptide RGDSP-FEFEFKFK (S: serine; P: proline), they developed a hydrogel scaffold that was used to inject rat cardiac progenitor cells (CPCs), a cell type known for its regenerative properties, into rat hearts. This study showed that culturing the CPCs inside the hydrogel for the duration of one week stimulated their differentiation towards adult cardiac phenotypes. The hydrogel injection containing the CPCs significantly reduced the left ventricular dilation and myocardial damage in rats after suffering an MI [111]. Recently, a new SAPH was developed by modifying RADA16, a peptide capable of self-assembling into β-sheets [114], with a cell-adhesive motif (RGDSP) and a BMP-2-binding motif [115]. The functionalized SAPH was tested for its effects on cell adhesion, differentiation, and survival of MSCs. When transplanted into rat myocardial infarction models, the SAPH formed nanofibrous scaffolds that supported cell adhesion and protected MSCs under hypoxic conditions. As the SAPH degraded, it led to sustained BMP-2 release, promoting MSC differentiation into cardiomyocytes. Four weeks after transplantation there was significant improvement in myocardial regeneration, angiogenesis, and cardiac function, along with increased cardiomyocyte differentiation and gap junction formation. This study suggests that SAPHs that enhance BMP-2 release can promote cardiac tissue regeneration [115].

### 4.6. Cardiac Patches

**Figure 2:**
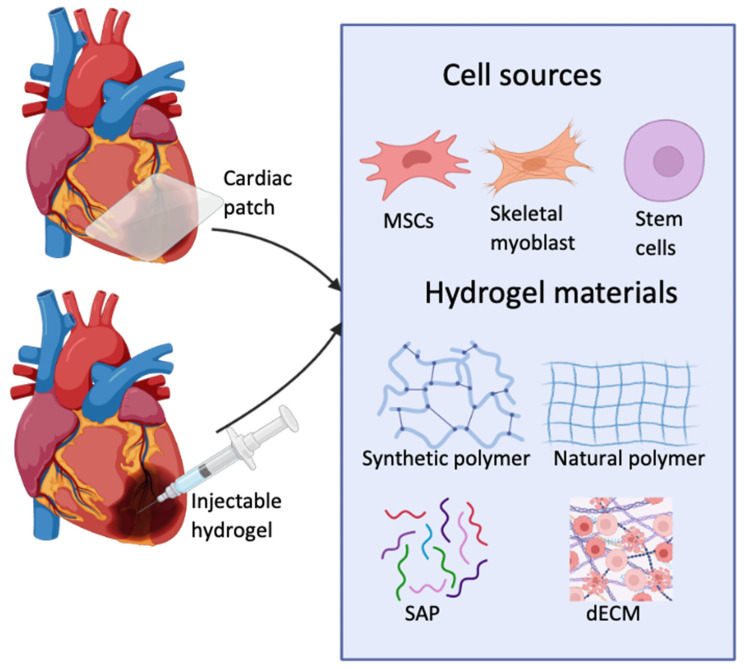
Application of cardiac patches and injectable hydrogels for repairing damaged heart tissue, along with examples of types of cells and hydrogel materials used in their development. Created with BioRender.com.

Cardiac patches are scaffolds engineered to adhere to the heart’s surface following MI damage [116] (Figure 2). They mimic the native ECM and provide mechanical support while promoting cardiac tissue regeneration. To be able to withstand the heart’s systolic pressure cardiac patches must maintain high mechanical properties [117]. Additionally, they must integrate seamlessly into the native cardiomyocyte’s electric conduction to prevent arrhythmias [118]. Patient-specific human cardiac patches have been created using hiPSC-CMs and decellularized rat heart ECM. These patches demonstrated contractility and normal electrical response and improved heart function in an MI rat model [119]. It was also noted that the hiPSC-CMs survived longer than those used in studies where they were injected directly into the heart [119,120]. In an MI porcine model, a combination of hiPSC-CMs, endothelial cells (ECs), and smooth muscle cells (SMCs) was co-injected, resulting in enhanced cell engraftment, improved myocardial wall stress, and contractility [121]. However, before cardiac patches can progress to clinical application, there are a number of obstacles that need to be overcome [122]. In a human heart, a cardiac patch needs to be of a certain size, large enough to cover the typical area of infarcted tissues. To address this, a cardiac patch with the appropriate dimensions of 4 cm × 2 cm × 1.25 mm was developed using hiPSC-CMs, SMCs, and ECs in a scaffold made from fibrin. In an MI porcine model, the patch significantly improved myocardial wall stress, decreased the size of the infarcted tissue area and improved overall cardiac function [123]. This shows the possibility of developing an effective cardiac patch with an increased size, that reflects the size required for a human heart.

Vascularisation is another major obstacle faced by researchers developing cardiac patches. It is essential as it provides the ability for the cardiac patch to exchange oxygen, nutrients, and signals with the host cells [122]. Recently, a partially vascularised cardiac patch was developed by fabricating biomimetic microvessels (BMVs) that contained human umbilical vein endothelial cells (HUVECs) and incorporated them into a fibrin gel that consisted of human cardiac stem cells (CSCs). These fabricated BMVs closely mimicked the architecture and function of natural capillaries. In a rat MI model, the BMV–CSC patch showed a significant improvement in myocardial capillary density and an uptake of cardiomyocyte mitotic activity in the infarcted area [124]. The BMV–CSC patch’s ability to generate vascularized tissue-engineered structures is a promising advancement for cardiac tissue engineering.

### 4.7. Injectable Hydrogels

Injectable hydrogels are widely used in cardiac tissue engineering due to their strong permeability, biocompatibility, and biodegradability [125] (Figure 2). Once injected into the heart, the hydrogel will change from liquid to a gel via crosslinking [126]. For example, thermosensitive hydrogels are engineered to develop into a gel at body temperature [89]. Similarly, ionic hydrogels transition from a liquid to a gel state in response to pH changes, while photo crosslinked hydrogels will solidify upon exposure to light, usually UV [126]. Research has demonstrated that using hydrogels that can be injected is an effective way to improve cardiac function and CM retention [125]. They can also increase the thickness of the left ventricle wall and offer support to the myocardium, which promotes tissue regeneration [127]. Injectable hydrogels have already been tested in clinical trials. An injectable hydrogel made of acellular alginate was investigated using 27 patients who had previously suffered an MI. Data from an echocardiograph revealed preserved LVEF, indicating that the left ventricle was functioning well. No adverse effects were recorded [128]. However, a larger clinical trial was conducted where they injected alginate-based hydrogels into patients with severe heart failure. The results demonstrated a significant reduction in all the participant’s overall HF symptoms alongside an increase in their exercise performance. Despite this, within a 30-day period after the injection, 8.6% of patients died, whereas in the control group, no patients died [129], emphasising the need to further investigate the safety and biocompatibility of injectable hydrogels.

**Figure 3:**
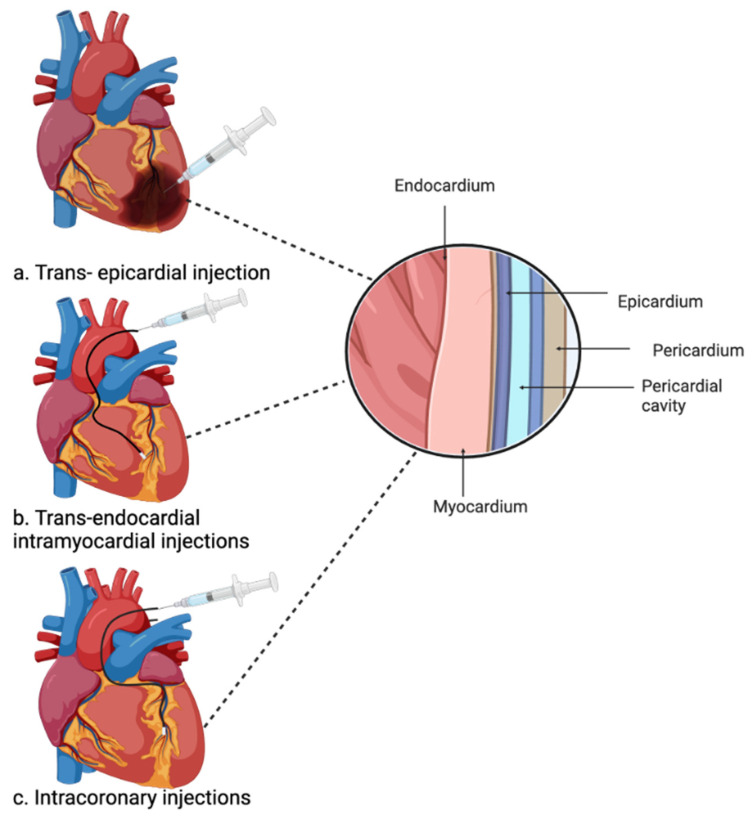
The three main administration methods of injectable hydrogels used in preclinical studies. (**a**) Trans-epicardial injection; (**b**) trans-endocardial intramyocardial injection; (**c**) intracoronary injections. Created with BioRender.com.

Injectable hydrogels hold significant promise in cardiac regeneration, but several challenges need to be addressed. These hydrogels often exhibit low mechanical strength and carry the potential to trigger an immune reaction upon injection into the heart [126]. Additionally, the application of injectable hydrogels for heart treatment aims to minimise the need for open heart surgery and enhance their clinical significance. However, the implantation methods themselves may pose certain risks. One common method involves directly injecting the hydrogel into the myocardium through the epicardium. This approach, however, necessitates a large injection, making it highly invasive for the patient, alongside a thoracotomy which introduces surgical complications [129,130] (Figure 3). Trans-endocardial intramyocardial injections use a catheter to guide the hydrogel to the heart injury site and are considered safer and more precise in terms of injecting the hydrogel into the myocardium through the endocardium using a catheter and imaging technology. Nevertheless, this method requires hydrogels with very specific mechanical properties, which limits the availability of suitable biomaterials [131,132]. Instead, intracoronary injection is delivered through the main coronary artery, but it carries the risk of coronary artery obstruction or re-embolization of coronary arteries, thus limiting its clinical use [100,128]. Despite the comparative safety and reduced invasiveness of injectable hydrogels as opposed to cardiac patches, a safer method for delivering hydrogels via injection is still needed to advance their clinical applications.

### 4.8. Current Progress of Organoids and 3D Bioprinting in Cardiac Regeneration

Organoids are scaled-down versions of organs and tissues, usually generated from stem cells, that create 3D structures which replicate to a degree the function and morphology of the original organ [133]. While these organoid constructs are still limited due to the complex nature of organs and tissues, they have proven effective in drug discovery, modelling diseases, and tissue engineering [132]. Scientists have successfully created cardiac organoid models, which have helped in understanding heart disease and provided a platform to study the biological mechanisms of human heart regeneration, bridging the gap between animal studies on MI and heart disease with human studies [134]. Cardiac organoids are still limited, due to their immaturity, lack of vascularisation, poor reproducibility, and a lack of structural features such as valves. As such, these organoids cannot fully replicate the adult human heart. Once these limitations are overcome, a variety of applications will be unlocked [135]. For instance, tissue generated from engineered cardiac organoids could potentially be transplanted into injured hearts post-MI to replace the damaged myocardium and restore cardiac function [136].

In the field of cardiac organoids, 3D bioprinting is a promising innovation that is gaining popularity. It involves the layering of biomaterials to create a 3D structure that is controllable, customisable, and reproducible [137]. A key component of this process is bio-ink, commonly made up of hydrogels derived from natural or synthetic polymers. A bio-ink should meet several important criteria to be suitable for 3D printing applications [138]. It must be biocompatible to support cell viability and integration with living tissues. Additionally, it should possess robust mechanical strength and flexibility to adapt to the diverse mechanical and chemical requirements of different tissue types [138,139]. Recently, advanced bioactive bio-inks have been developed, incorporating cells such as adult stem cells or iPSCs within them prior to printing. This allows for a more precise distribution of cells within the 3D structure [138,139,140].

**Figure 4:**
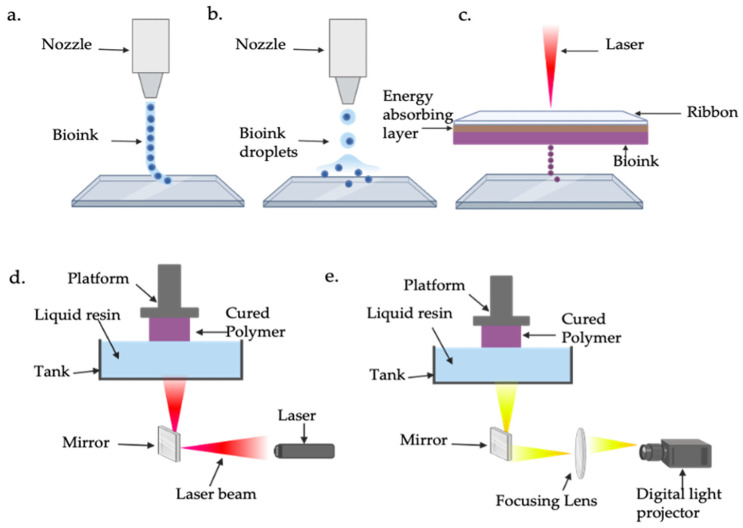
Schematic representation of bioprinting techniques. (**a**) Extrusion based; (**b**) droplet based; (**c**) laser assisted; (**d**) stereolithography; (**e**) digital light processing. Created with BioRender.com.

While 3D bioprinting has been effective in tissue engineering for hard tissues such as bone, it is still limited regarding soft materials such as hydrogels and more complete structures like the heart [141]. There are four main bioprinting techniques that vary based on their mechanisms, applications, and resolutions (Table 4) (Figure 4). Droplet-based bioprinting employs bio-printers that continuously deposit biomaterials, bio-inks, or cell-laden droplets to construct 2D or 3D structures. It has high precision, achieving resolutions of up to 50 μm, but struggles with printing highly viscous bio-inks [142,143]. Laser-assisted bioprinting uses a high-intensity laser to propel biomaterial droplets onto a substrate containing cell culture medium. This method requires a ribbon composed of transparent glass, a thin metal layer, and a biomaterial layer, where laser pulses vaporise the metal layer, generating a high-pressure bubble to eject droplets with exceptional precision, achieving resolutions as fine as 5 μm [144,145]. Stereolithography (SLA) and digital light processing (DLP) rely on light-sensitive polymers to fabricate intricate structures. These techniques use either a UV laser beam (SLA) or digital light sources like UV (DLP) to cure liquid photocrosslinkable bio-inks, enabling the creation of highly detailed constructs [146]. Finally, extrusion-based bioprinting stands out as the most commonly used technique due to its simplicity, affordability, and scalability. It employs a computer-controlled system to extrude bio-inks or biomaterials through a nozzle using a pressure system, creating 3D structures layer by layer [147]. While its resolution, at approximately 200 μm, is lower compared to other methods, extrusion-based bioprinting is highly versatile, capable of printing higher cell densities and more viscous bio-inks, and offers better structural integrity through the continuous deposition of filaments. Despite its limitations, extrusion-based bioprinting remains the most popular method due to its practicality in producing larger-scale constructs [148]. In addition, the Freeform Reversible Embedding of Suspended Hydrogels (FRESH) is an emerging extrusion-based bioprinting technique, where hydrogel bio-inks are extruded within a thermo-reversible support medium. This technique aims to address challenges faced when developing soft, complex structures such as the heart [149]. A study introduced an optimised version of the bioprinting technique called FRESH v2.0, which significantly enhanced resolution, enabling the creation of collagen filaments with a resolution of 20 μm in diameter. Using this advanced approach, researchers successfully bioprinted a neonatal-scale human heart using type I collagen bio-ink as a scaffold. To allow for the observation of internal structures, they printed only half of the heart, showcasing intricate features such as trabeculae, atrial and ventricular chambers, and pulmonary and aortic valves [150]. This highlights how bio-inks and advanced 3D bioprinting techniques, such as extrusion-based bioprinting, can be used to generate functional cardiac tissue, offering a promising approach for advancing research in cardiac regeneration and developing effective therapies to repair and regenerate heart tissue damaged by MI.

## 5. Concluding Remarks and Future Perspectives

### 5.1. Hydrogel Composition

There is an urgent clinical need for effective novel therapies to treat MI. Various cell types, such as adult stem cells and pluripotent stem cells, show potential for therapeutic application [151]. However, their success depends on the development of suitable scaffolds that can support cell growth and differentiation while also ensuring biological compatibility. Hydrogels offer a promising possibility as a scaffold to support cells in the regeneration of damaged cardiac tissue after an MI [152]. Researchers have faced challenges in developing hydrogels tailored specifically for heart regeneration [55], since they must be meticulously designed to accommodate the mechanical and electrical properties of the heart while also being able to withstand the strong contractions generated by the cardiac muscle [103].

Natural-based hydrogels have proven to be effective in cardiac regeneration due to their ability to mimic the ECM, their biocompatibility, biodegradability, and their very low cytotoxicity, making them suitable for clinical application [153]. Unfortunately, they possess low stability, weak mechanical strength, and rapid degradation in vivo [154]. Conversely, synthetic hydrogels have more controllable features and strong mechanical properties, which provide structural support for the cells, but they have poor biocompatible and biodegradation properties. Some synthetic polymers can be modified through alteration of their functional groups or by combining them with natural polymers to improve their weaker biocompatibility [155], and the development of these hybrid hydrogels along with the incorporation of conductive nanomaterials has led to a significant advancement in cardiac tissue regeneration.

### 5.2. Hydrogel Delivery Methods

Hydrogels in cardiac tissue regeneration have various delivery methods. Injectable hydrogels are an innovative, minimally invasive approach. They respond to temperature, light, and pH [156], becoming more adaptable after injection. Moreover, incorporation of conductive materials further improves their electrical integration [104]. The advancement of injectable hydrogels has been revolutionary in cardiac tissue regeneration; however, they are affected by poor mechanical force transmission [89] which is being addressed by shear thinning, a technique resulting in increased mechanical strength and stiffness, as well as improved self-healing properties [157]. Nevertheless, to further develop the regenerative capabilities of injectable hydrogels, more novel studies are needed to address the injection application, degradation rate, low survival, and the retention of therapeutic cells.

Hydrogel-based cardiac patches also show promising potential in cardiac tissue repair, especially after an MI. Once integrated with the host, they can deliver therapeutic cells to repair damage in the myocardium. However, limited full electrical integration with the damaged myocardium, immune rejection, and potential surgical complications during transplantation to the heart [116,117,158] limit their therapeutic application. Notably, a novel injectable conductive cardio patch was developed using methacrylated gelatin and elastin, along with CNTs. The remarkable feature of this patch is that it can re-form its original shape post-injection. Tests in rats and minipigs demonstrated significant improvement in cardiac function post-MI [159]. More importantly, this study utilised CNTs to produce cardiac patches that were electrically conductive, a feature not seen in previous research [159]. This breakthrough can lead to the development of an injectable minimally invasive cardiac patch with improved conductive properties. This further highlights the possibilities an injectable cardiac patch could have for post-MI treatment in a clinical setting.

### 5.3. Current and Future Prospects of Bioprinting in Cardiac Regeneration

Bioprinting has emerged as an innovative technology with the ability to create and develop 3D structures made from various biomaterials. Its potential applications have garnered significant attention, particularly when combined with stem cells [138,160,161]. The ability to fabricate heart tissue through bioprinting holds promise for addressing heart disease and tissue damage, but there are still numerous challenges that must first be addressed and overcome to ensure its success in clinical applications. These obstacles involve the printing techniques, the bio-inks, the mechanical strength of printed 3D structures, cell viability, and vascularisation. In addition, the bio-inks also need improvements in compatibility, stiffnesses, integrin signalling, and cell motility [162]. A promising area of future research in bioprinting is 4D bioprinting, an advanced technique that combines 3D bioprinting technology with smart materials capable of changing their properties or shapes in response to external stimuli. These materials are engineered to react to factors such as pH, light, temperature, or electric and magnetic fields, enabling dynamic and responsive changes in the printed structures over time [163]. In 4D bioprinting, hydrogels play a crucial role as they can be designed to undergo these stimuli-driven changes, making them particularly suitable for biomedical applications. This innovative approach is especially significant in cardiac regeneration, where it enables the creation of scaffolds that can adapt to the heart’s dynamic environment, respond to biomechanical cues, and promote tissue growth and repair [164]. By mimicking the heart’s native behaviour and adapting to its changing conditions, 4D bioprinting offers the potential for advancing research in cardiac regeneration and improving therapies for MI [161].

In summary, synthetic and natural hydrogels possess unique qualities that can provide a supporting environment, enhancing the differentiation and maturation of implanted cells. Hydrogels can be used in various applications such as cardiac patches, injectable hydrogels and 3D bioprinting, which can help promote cardiac tissue regeneration. Although these methods have great potential, some refinement is needed before proceeding to clinical applications.

## Figures and Tables

**Table 1:** Types of cells used in cardiac regenerative research.

Cells Type	Origins	Pros	Cons	References
Skeletal myoblasts	Progenitor cells located within skeletal muscle.	Rapidly divide Resist ischemia	Limited electrical integration Do not fully differentiate into cardiomyocytes Increased risk of ischemia	[25,26]
Mesenchymal stem cells	Multipotent cells originate from various tissues/organs, such as bone marrow and adipose tissue.	Anti-inflammatory effects Easy isolation and expansion	Low cell retention Limited cell engraftment and survival	[26,27]
Embryonic stem cells	A pluripotent stem cell that originates from in the inner cell mass of human blastocysts, at early stages of the embryo’s development.	Pluripotency High proliferation	Risk of tumour formation Ethical concerns Higher risks of immune rejection	[28,29,30]
Induced pluripotent stem cells	A pluripotent stem cell that can be generated directly from a somatic cell using specific growth factors.	Pluripotency Patient specific High proliferation No ethical concerns	Immature differentiation and maturation Risk of tumour formation High costs	[31,32,33]

**Table 2:** Natural hydrogel-forming polymers.

Polymers	Attributes	Pros	Cons	References
Collagen	Provides structural support to cardiac cells and contributes to the heart’s stiffness and rigidity. Assists in force transmission ensuring efficient force is generated by the heart to pump blood around the body.	Biocompatible Biodegradable Incorporated with other polymers to enhance its physical properties	Unstable Poor mechanical properties	[19,24,81,82]
Gelatin	Partially hydrolysed from collagen Functions as a crosslinking polymer, imparting essential properties like structure and texture to the gel. Retains the arginine-glycine- aspartic acid (RGD) peptide sequence which promotes cell adhesion, proliferation, and differentiation.	Non-immunogenic Biocompatible Biodegradable	Low thermostability Weak mechanical strength	[27,83,84]
Hyaluronic acid (HA)	Natural, linear polysaccharide that contains multiple acid and hydroxyl groups chemically modifiable to improve its mechanical properties.	Biocompatible Biodegradable Non-immunogenic Non-thrombogenic	Weak mechanical strength High degradation rate	[85,86]
Elastin	Provides elasticity to various tissues and organs Tropoelastin is a soluble precursor of elastin that has similar biochemical properties to elastin.	Can be hydrolysed to form smaller chains such as peptides and polypeptides, which are soluble	Insoluble nature Weak mechanical properties Batch-to-batch variation	[62,87,88]
Chitosan	Linear polysaccharide attained by the partial deacetylation of chitin.	Low toxicity Antibacterial properties Encourages angiogenesis	Weak mechanical properties Batch-to-batch variation	[62,89]
Fibrin	Plays a role in the body’s natural tissue repair mechanism.	Biologically compatible Ability to promote repair Increased survival rate of CMs	Poor mechanical strength Hydrogel shrinkage Batch to batch variation	[87,89,90]
Decellularized ECM (dECM)	Developed through decellularization, which involves the elimination of cells from the ECM to generate a natural matrix.	Supports cell growth, adhesion, and remodelling Non-immunogenic	Poor mechanical properties Cytotoxicity Batch-to-batch variation	[89,91,92]

**Table 3:** Synthetic hydrogel-forming polymers.

Synthetic Polymers	Attributes	Advantages	Disadvantages	References
Polyacrylamide (PAA)	Developed via crosslinking of acrylamide monomers.	Stable Non-toxic Hydrophilic Highly adaptable	Short lifespan, which limits cell culture studies	[93,94,95]
Polyethylene glycol (PEG)	FDA-approved water-soluble polymer. Modifying this hydrogel with an RGD peptides improves cell proliferation and survival.	Biocompatible Non-immunogenic Strong mechanical properties	Not soluble Inadequate cell specific adhesion	[80,96,97]
Poly-N-isopropyl acrylamide (PNIPAAm)	Changes from liquid to gel above temperatures over 32 °C.	Thermo-responsive behaviour	Weak mechanical strength Low biodegradability	[89,91,98]
Polyvinyl alcohol (PVA)	Developed via the hydrolysis of polyvinyl acetate. As a hydrogel, it has high elasticity and can enhance the dispersion of mechanical signals.	Hydrophilic Biocompatible Biodegradable	Low adhesion Low biodegradability Poor thermo-responsive behaviour	[80,89,99]

**Table 4:** 3D bioprinting techniques.

Bioprinting Technique	Pros	Cons	References
Extrusion-based	Not complex Affordable Scalable	Cell damage through sheer stress Limited available biomaterials Low resolution Clogging of the nozzle	[147]
Droplet-based	Affordable Accessible High cell viability >90% High resolution	Limited available biomaterials Issues with fabricating porous tissue structures Clogging of the printer’s injector	[143]
Laser-assisted	Nozzle free High resolution Wide range of available biomaterials High cell viability Low mechanical stress	High cost Time consuming	[145]
Stereolithography (SLA) and Digital Light Processing (DLP)	High resolution High speed High cell viability Nozzle free	Cytotoxic effects Limited available bio-inks	[146]
